# Acute Effects of Walking and Standing on Executive Function in Children with Attention Deficit/Hyperactivity Disorder: A Feasibility Study

**DOI:** 10.3390/children11030341

**Published:** 2024-03-13

**Authors:** Amanda Barudin-Carreiro, Sarah M. Camhi, Heidi I. Stanish, Julie A. Wright

**Affiliations:** 1 Counselor Education Department, Bridgewater State University, Bridgewater, MA 02325, USA; abarudincarreiro@bridgew.edu; 2 Department of Kinesiology, University of San Francisco, San Francisco, CA 94117, USA; 3 Exercise and Health Sciences Department, University of Massachusetts Boston, Boston, MA 02125, USA; heidi.stanish@umb.edu (H.I.S.); julie.wright@umb.edu (J.A.W.)

**Keywords:** ADHD, executive function, exercise, physical activity, feasibility

## Abstract

Children with attention deficit/hyperactivity disorder (ADHD) struggle with executive functioning (EF). While physical activity (PA) benefits EF, little is known about the impact of specific activities like standing. The purpose of this study was to evaluate the feasibility of performing a rigorous experimental study to compare the effects of walking and standing on EF in children with ADHD. Six areas of feasibility were assessed: recruitment, randomization, treatment adherence, retention, acceptability of the intervention, and implementation. A randomized pilot study using three parallel conditions compared the effects of two modes of activity on EF in children 6–11 with ADHD. While there were no significant differences between walking and standing for EF, analyses suggest that it is feasible to compare effects of standing vs. walking on EF among children with ADHD. This study supports the feasibility of undertaking a larger scale study to evaluate the effect of standing on EF in children with ADHD.

## 1. Introduction

Attention deficit/hyperactivity disorder (ADHD) is the most common neurodevelopmental disorder of childhood, with males more frequently diagnosed than females [1,2,3]. The prevalence of ADHD in children 2 to 17 years is estimated at 9.4% in the United States [4] and worldwide estimates of its prevalence in children under 18 are 7.2% [5].

The reduced blood flow to the prefrontal and frontal regions of the brain associated with ADHD causes dysfunction in executive functioning (EF), which involves attention, working memory, response inhibition and planning [6,7,8,9]. There is no universally accepted definition for EF; however, the generally agreed upon core components consist of inhibition (inhibitory control, self-control/behavioral inhibition) and interference control (selective attention and cognitive inhibition), working memory (which can be verbal and nonverbal) and cognitive flexibility (commonly called set shifting) [10,11,12].

Based on current reviews and meta-analyses, there appears to be preliminary evidence showing that PA is associated with improvements in EF in children with ADHD [13,14,15,16]. fMRI studies have indicated that there is decreased blood flow to the prefrontal areas of the brain in children with ADHD [6,8,9], suggesting that PA could serve as a potential mechanism by which to promote more cerebral blood flow, which in turn could improve inattention, hyperactivity, and impulsivity deficits found in this population.

Although studies suggest that physical activity (PA) generally improves EF in children with ADHD, the quality of research is low due to poorly described study protocols (i.e., unclear about randomization, blinding), variability in outcome assessments, and a high risk of bias, such as selection bias, detection bias, and performance bias [7,13,14,15,16,17,18,19,20,21,22,23]. Little is known about the intensity, duration, and type of PA needed to have a positive impact on EF. Studies have found acute bouts (15 to 20 min) of moderate intensity exercise have a significant impact on inhibition [24,25,26] and cognitive flexibility [26,27] compared with a sedentary control.

While there is preliminary evidence suggesting that moderate intensity PA positively influences EF, less is known about lighter intensity activities, such as standing. Data suggest that around 60% to 65% of a child’s waking hours are spent sedentary, either in school or at home, and with advances in technology and environmental changes, sedentary behavior is becoming more prevalent [28,29,30,31,32]. The use of standing desks in classroom settings has been shown to facilitate learning, improve attention, working memory, and cognitive flexibility in children without ADHD [29,30,31,33,34,35,36,37,38,39,40]. Yet to our knowledge, no studies have examined the effect of standing on EF in children with ADHD.

Rigorous experimental studies require time, effort, and resources [41]. Before moving to a randomized, controlled trial, pilot studies are a necessary first step in exploring the feasibility of recruitment, randomization, intervention implementation, assessment procedures, and retention [42]. The purpose of this study is to evaluate the feasibility of examining the effects of standing on EF performance in children (ages 6 to 11) with ADHD and comparing the effects of standing and walking in this population. Evaluating the feasibility of this study based on recruitment of the sample, randomization protocol, treatment adherence, retention of the sample, acceptability of the intervention, and implementation, the potential for a full-scale study will be determined [42,43,44,45].

## 2. Materials and Methods

### 2.1. Design and Procedures

A randomized experimental pilot study using three parallel conditions (groups) was used to assess EF performance in children aged 6 to 11 years with a parent-reported diagnosis of ADHD. Participants were recruited between May 2019 and May 2020 via mental health agencies, independent counselor offices, schools, pediatrician offices, and social media sites (i.e., the CHADD website, Facebook Event, Craigslist). Eligibility was assessed via a telephone screening. Verbal parental consent for eligible participants was obtained prior to scheduling an in-person visit. Eligible participants and their parent/guardian were invited to attend a one-time visit where written parental consent and child assent were obtained. Height and weight were collected to calculate body mass index (BMI kg/m^2^) and the Stroop Color–Word Test—Children’s Version (SCWT) and the Wisconsin Card Sorting Task (WCST) were administered pre and posttest to assess EF. A coin toss determined the order of administration for the SCWT and WCST. After the pretest assessments were completed, a sealed envelope was used to randomize the child to one of the following three conditions: (a) walking (b) standing, or (c) sitting for 20 min. Participants in each condition were given an iPhone and earbuds to listen to an age-appropriate music playlist created by the first author. HR was collected as a descriptive measure and was monitored every minute for 20 min in all conditions. Parents waited in a separate room during the testing sessions. Participants received a $50 gift card, and the parent received a list of publicly available resources of the benefits of exercise and ADHD. The study was conducted according to the guidelines of the Declaration of Helsinki and approved by the Institutional Review Board of University of Massachusetts Boston (protocol 2019060).

### 2.2. Participants

Participants were children (a) between 6 and 11 years old, (b) with a parent-reported diagnosis of ADHD as established by a trained psychologist, psychiatrist, licensed mental health counselor, social worker, or medical doctor, and (c) English speaking. Participants were excluded if they (a) were younger than 6 or older than 11; (b) could not walk, stand or sit unassisted for 20 min; (c) had a comorbid mental health diagnosis (anxiety, obsessive-compulsive disorder, depression, oppositional defiant disorder, intermittent explosive disorder, conduct disorder, reactive attachment disorder, autism spectrum disorder, post-traumatic stress disorder); (d) were taking medication for a condition other than ADHD (i.e., antipsychotics, antidepressants, mood stabilizers, anti-anxiety/anxiolytics, medication for sleep, medication for seizures); or (e) were non-English speaking.

### 2.3. Demographics

A parent self-report questionnaire was used to obtain demographic characteristics such as age, sex, race/ethnicity, grade level, medication use, ADHD subtype, co-occurring learning disorder, family history of ADHD, parental/guardian marital status, annual family income, and highest grade level completed by the parent/guardian.

### 2.4. Physical Activity

A question from the 2017–2018 NHANES Physical Activity and Physical Fitness Questionnaire (PAQ) [46] was used to estimate PA levels based on proxy report from the parent/guardian. The proxy respondent was asked “During the past 7 days, on how many days was {child’s name} physically active for a total of at least 60 min per day? Add up all the time {child’s name} spent in any kind of physical activity that increased {his/her} heart rate and made {his/her} breathe hard some of the time”. Response range was from 0 to 7 days.

### 2.5. Heart Rate

Heart rate (HR) was measured with a Polar H7 Bluetooth heart rate monitor (Polar, USA) to assess intensity level of PA throughout the intervention period.

### 2.6. Anthropometrics

Participant’s height (cm) and weight (kg) were measured with shoes on using a wooden stadiometer and a calibrated Health o Meter Professional digital scale (Pelstar, Countryside, IL, USA). BMI (kg/m^2^) was calculated and BMI percentiles for age and gender were determined using the CDC’s BMI Percentile Calculator for Child and Teen [47].

### 2.7. Stroop Color and Word Test Children’s Version

The SCWT assesses inhibition/inhibitory control. The test can be used for children 5 to 14 years of age, with specific administration, scoring, and interpretive strategies for use with younger children (ages 5 to 10 years) and older children (ages 11 to 14 years). The validity of the test has been reported to be between 0.80 and 0.91 [48].

The SCWT Children’s Version requires participants to read three different tables as fast as possible in 45 s. The first two tables represent the congruous conditions (word condition and color condition). The third condition represents the incongruent condition, named the color word condition and in which color words are printed in an inconsistent color ink. Participants were instructed to name the color of the ink instead of reading the word (i.e., the word “blue” is printed in green ink). The test yields 3 scores (raw word score, raw color score and raw color word score), based on the number of items completed. The raw scores are converted to *t* scores by age. Age adjusted *t* scores for the color word task were used in the present study.

### 2.8. Wisconsin Card Sorting Task

The WCST measures problem solving and cognitive flexibility/shifting. More specifically, it measures planning, updating, modulating impulsive responding and perseveration [49]. This task requires the ability to develop and maintain a problem-solving strategy across changing stimulus conditions to achieve a goal. The WCST is validated for use with individuals between 6 to 89 years of age with interscorer reliability coefficients ranging from 0.895 to 1.000 and intrascorer reliability coefficients ranging from 0.828 to 1.000 for the 11 scores of the WCST [50]. The WCST is also considered a valid measure of EF in clinical groups, such as children with ADHD [49,51].

The test used four key cards and 128 response cards. The participant was presented with four key cards and two decks of 64 response cards. They were asked to match the response cards to the key cards but were not told in what way the response cards should match. The researcher provided “right” or “wrong” feedback after each response trial. The task was terminated by either successful completion of all six categories or until all cards in both decks had been used (the end of the 128 response cards). For the present study, age-adjusted *t* scores were calculated for total errors, perseverative response, perseverative errors, non-perseverative errors, and percent conceptual level response. *T* scores in the 45–54 range are in the average range and *t* scores 55 or greater are in the above-average range [51].

### 2.9. Data Analysis

Criteria used to evaluate feasibility is outlined in Table 1. Data were cleaned by checking data entry for 20% of total participants. Means, standard deviations (SD), percentages, and frequencies were used to describe the sample’s characteristics. Change scores from pretest to posttest were computed for EF measures using the formula posttest minus pretest. One way analysis of variance (ANOVA) was used to compare between group mean scores and change scores. ANOVA tests were followed with a Kruskal–Wallis test for nonparametric data, in order to examine the change scores for the SCWT and five categories of the WCST (total errors, perseverative responses, perseverative errors, non-perseverative errors, percent conceptual level responses). Percent change scores were also computed for primary outcomes in order to explore how much change the walking, standing, and sitting group had from pretest to posttest. IBM SPSS version 27 was used for the analyses. Alpha level was set at *p* < 0.05.

## 3. Results

### 3.1. Recruitment of the Sample

**Figure 1:**
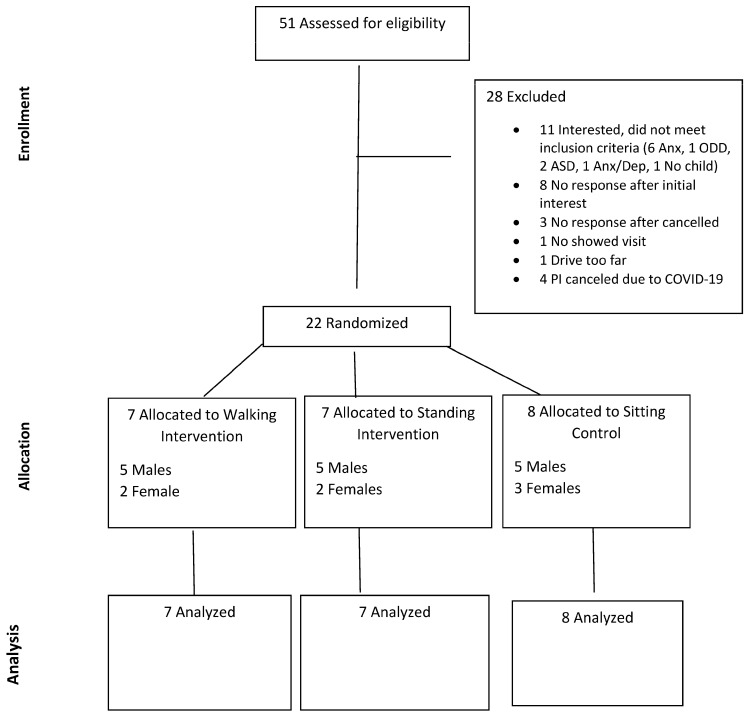
Diagram of sample selection process for the study.

Figure 1 presents the enrollment, allocation, and analysis data for the study. Of the 51 potential participants assessed, 22 (43%) met eligiblity and completed the study. Due to COVID 19 restrictions, four (8%) of the initial respondents who were screened for eligilbity and scheduled for a visit for data collection were cancelled. Table 2 displays participant characteristics.

### 3.2. Randomization Protocol

All participants were randomized via block randomization to ensure equal numbers in each condition.

### 3.3. Measurement and Retention Rates

All 22 participants who attended their study visit completed the measurement protocol. No participant dropped out during measurement, suggesting that the measurement and treatment protocol was acceptable. The one-time visit had a 100% retention rate of participants once they began the in-person measurement protocol. Due to the COVID 19 pandemic, the study ended early and our aim to have equal numbers in each condition was not achieved.

### 3.4. Acceptability of the Intervention

No complaints were received from participants who completed the measurement protocol. During the screening process there was one parent who felt the drive was too far and was no longer interested in having their child participate. One participant failed to attend their scheduled visit and did not respond to reschedule, and three parents cancelled their scheduled visits, due to unreported reasons, with no response to reschedule.

During the intervention there were times when children would ask “how much longer do I have to walk/stand/sit”; however, as a whole, children did not report that they disliked the intervention or asked to stop the study early.

### 3.5. Treatment Adherence and Implementation

Participants completed all stages of data collection and parents filled out what they were asked to complete.

### 3.6. Inhibition

There were no significant differences between the three groups. Table 3 presents the pretest score, posttest score, and change score from pre to posttest for the three conditions. Walking had a lower change score when compared with standing and sitting. Table 4 presents the percent change scores. The sitting group had the largest percent change in inhibition when compared with standing and walking.

### 3.7. Cognitive Flexibility/Shifting

One way ANOVA and the Kruskal–Wallis test revealed no significant differences in any of the five categories of the WCST. Table 4 displays the percent change. Although not statistically significant, standing had the highest percent change on all five categories of the WCST when compared with walking and sitting. Mean change scores, although not significant, also indicate that walking and standing improved performance more when compared with sitting, with standing showing the most improvement on all WCST categories (see Table 5).

### 3.8. Heart Rate

One way ANOVA showed significant between group differences (*F*(2,19) = 14.46, *p* < 0.001). Tukey post hoc analyses revealed significant pairwise differences in HR between walking and sitting. Walking resulted in 30 more beats/min (*p* < 0.001) compared with sitting and 27 more beats/min compared with standing (*p* = 0.001). Standing HR and sitting HR were not different (*p* > 0.05).

## 4. Discussion

Previous studies have not evaluated the feasibility of implementing a rigorous study design to examine the effect of standing on EF in children with ADHD. The findings of this study suggest that it is feasible to further explore the effect of standing on EF and to compare the effects of acute bouts of walking and standing on EF in children ages 6 to 11 years with ADHD. Recruitment was successful, despite ending early due to the COVID 19 pandemic. Randomization was successfully implemented and there was a 100% retention rate, suggesting that, due to the limited burden placed on families, with only one visit being required, all aspects of the measurement protocol were completed by participants.

Due to the small sample size, the study was insufficiently powered, and results should be interpreted with caution. Although there were no significant differences, the present study implemented a more rigorous study protocol when compared with previous research [24,25,26,27,52,53,54,55]. In the present study a control group was used, randomization was implemented, counterbalancing was employed to reduce carryover effects from the participants taking the same assessment at pre and posttest, all assessments were completed, and the study was conducted in a laboratory setting, all of which increased internal validity. Common, valid outcome measures were also used to allow the results to be compared with previous studies.

Previous studies have not examined the effects of standing desks on EF in children with ADHD; however, improvement in inhibition and cognitive flexibility/shifting in children without ADHD has been reported after standing at a desk for 20 to 60 min [28,29,30,31,33,35,36,40,56]. Although inhibition and cognitive flexibility were not significnatly improved after standing, the trends observed appear to be in line with previous research that examined standing desks and EF in children without ADHD [28,29,33,35,36,37,39,40]. Additionally, the trends observed appear to be in accordance with findings that suggest that PA could serve as a mechanism by which to promote more cerebral blood flow [6,8,9]. Exploring the effect of standing on EF in this population may provide options and alternatives with which to enable this population to become more successful in various aspects of day-to-day life, such as improved impulse control, attention, on-task behavior, and might serve as a potential option for schools to help improve academic performance in children with ADHD.

Although the current study has many strengths there are several limitations that need to be considered. Because the sample size was small, Type II errors cannot be ruled out and findings should be interpreted cautiously. Though the study did not derive any significant results, replication of the study protocol with a larger sample will allow for a more thorough exploration into the effects that walking and standing may have on EF in children with ADHD. The study was conducted by only one researcher, so the possibility of performance and detection bias cannot be ruled out. However, to try to mitigate this limitation and to not bias the pretest assessment process, randomization of conditions was achieved by placing them in a sealed envelope that was not opened until after pretest assessments were completed. The use of valid outcome measures, with standardized administration, interpretation, and scoring, was also utilized to minimize any researcher bias. As participants completed the same assessments twice in a short period of time, testing effects and fatigue could have occurred. The study was also ended early due to the COVID 19 pandemic, which reduced the sample size.

Future research that can build upon this feasibility study is recommended. Recruiting a more diverse sample size, using various settings, such as schools or community-based programs, modifying the pre and posttest interval time frame, and adding more objective PA measures would increase the generalizability of the findings.

## 5. Conclusions

By determining that the study design was feasible for examining the effect of standing on EF in children with ADHD, conducting more rigorous, larger scale studies will add to the literature and allow for a better understanding of how walking and standing impact EF in children with ADHD. Children spend most of their day at school, where some schools reduce recess and physical education to increase the amount of standardized testing and lecture time spent in the classroom, which promotes a high volume of sitting throughout the day [29,33,57]. Further examination of the effect of standing on EF in this population can be important for policy changes, especially within school systems, and will help to provide parents, school personnel, providers, and other stakeholders additional options and recommendations to address this public health concern and treat this diagnosis across multiple settings.

Recent studies have started to examine PA as a possible alternative and/or adjunct treatment option for this population; however, more sophisticated research needs to be undertaken and there is insufficient evidence to support PA as a stand-alone treatment [58]. Rigorous studies are needed to further pave the way for more consistent, clinically relevant options to be made available to educators, parents, and stakeholders so as to better address this public health concern within this population [17,59].

Results from the present study also have the potential to help guide policy considerations, especially within school systems, as they appear to be in line with previous research that has concluded that standing is an acceptable way to promote more movement in children, especially as they spend most of their day sedentary, without distracting from the focus of teaching [28,29,30,31,33,34,37,38]. Results from larger scale replication studies could offer more definitive recommendations for improving EF in children with ADHD.

## Figures and Tables

**Table 1:** Criteria used to evaluate the feasibility of the study.

Area of Feasibility	How Area Is Evaluated
Recruitment of the sample	The number of participants screened per month; number enrolled per month
Randomization protocol	Participants are willing to be randomized
Measurement protocol	Assessment completion rate at the end of the study
Retention of the sample	The number of participants in each group who remained in the study
Acceptability of the intervention	The level of satisfaction and perceived appropriateness to continue as assessed by those who delivered and received the intervention
Implementation	The rate of delivery to those enrolled

Note. The data in column two are adapted from the following sources [42,43,44,45].

**Table 2:** Demographic characteristics of the sample across the three conditions (*n* = 22).

Variables	Walking (*n* = 7) M (SD) or *n (*%)	Standing (*n* = 7) M (SD) or *n (*%)	Sitting (*n* = 8) M (SD) or *n (*%)	Total (*n* = 22) M (SD) or *n (*%)
Age (years)	9.69 (1.75)	9.18 (1.46)	9.65 (1.19)	9.51 (1.42)
Range	6.42–11.50	6.92–11.25	8.33–11.92	6.42–11.92
Median	10.25	9.42	9.25	9.59
Sex				
Male	5 (71.4%)	5 (71.4%)	5 (62.5%)	15 (68.2%)
Female	2 (28.6%)	2 (28.6%)	3 (37.5%)	7 (31.8%)
Race				
White	5 (71.4%)		7 (87.5%)	19 (86.4%)
Black	1 (14.3%)	7 (100%)	1 (12.5%)	2 (9.1%)
Hispanic/Latino	1 (14.3%)			1 (4.5%)
Grade Level	4 (2.16)	3.43 (1.27)	4.13 (.99)	3.86 (1.49)
Range	K-6	2–5	3–6	K-6
Median	5	3	4	4
				
Kindergarten	1 (14.3%)	0	0	1 (4.5%)
First	0	0	0	0
Second	1 (14.3%)	2 (28.6%)	0	3 (13.6%)
Third	0	2 (28.6%)	2 (25%)	4 (18.2%)
Forth	0	1 (14.3%)	4 (50%)	5 (22.7%)
Fifth	4 (57.1%)	2 (28.6%)	1 (12.5%)	7 (31.8%)
Sixth	1 (14.3%)	0	1 (12.5%)	2 (9.1%)
BMI * (Percentile)	57.29 (38.51)	54.71 (29.10)	56.38 (30.18)	56.14 (31.12)
Range	1–98	6–97	7–98	1–98
Median	39	59	59	59
				
Underweight (≤5)	1 (14.3%)	0	0	1 (4.5%)
Normal weight (5–84)	3 (42.9%)	6 (85.7%)	6 (75%)	15 (68.2%)
Overweight (85–94)	1 (14.3%)	0	1 (12.5%)	2 (9.1%)
Obese (≥95)	2 (28.6)	1 (14.3%)	1 (12.5%)	4 (18.2%)
Number of days past week active 60 min	5 (1.13)	4 (1.60)	4 (1.31)	5 (1.44)
Range	4–7	2–7	2–6	2–7
Median	5	4	4	5
				
Met PA Guidelines	2 (28.6%)	1 (14.3%)	0	3 (13.6%)
Did not meet PA Guidelines	5 (71.4%)	6 (85.7%)	8 (100%)	19 (86.4%)
ADHD Type				
Inattentive	2 (28.6%)	1 (14.3%)	2 (25%)	5 (22.7%)
Impulsive/Hyperactive	2 (28.6%)	0	0	2 (9.1%)
Combined	2 (28.6%)	6 (85.7%)	6 (75%)	14 (63.6%)
Unknown	1 (14.3%)	0	0	1 (4.5%)
ADHD Medication Use	5 (71.4%)	3 (42.9%)	6 (75%)	14 (63.6%)
Family history ADHD	3 (42.9%)	4 (57.1%)	6 (75%)	13 (59.1%)
First degree family member (1 Member)	1 (14.3%)	3 (42.9%)	2 (25%)	6 (27.3%)
First degree family member (2 Members)	1 (14.3%)	0	3 (37.5%)	4 (18.2%)
No first-degree family member	4 (57.1%)	4 (57.1%)	3 (37.5%)	11 (50%)
Unanswered	1 (14.3%)	0	0	1 (4.5%)
Learning D/O	1 (14.3%)	1 (14.3%)	1 (12.5%)	3 (13.6%)
Unknown			1 (12.5%)	1 (4.5%)
Household income				
<$75,000	1 (16.7%)	2 (28.6%)	3 (42.9%)	6 (27.3%)
75,000 and above	5 (83.3%)	5 (71.4%)	4 (57.1%)	14 (63.6%)
Unanswered	1 (14.3%)		1 (12.5%)	2 (9.1%)
Marital status				
Single	1 (14.3%)	0	2 (25%)	3 (13.6%)
Married	6 (85.7%)	6 (85.7%)	6 (75%)	18 (81.8%)
Divorced	0	1 (14.3%)	0	1 (4.5%)
Highest level of education of family member				
≤HS ^a^/equivalent	0	2 (28.6%)	1 (12.5%)	3 (13.6%)
≥BA ^b^ degree	5 (23.8%)	4 (57.1%)	5 (62.5%)	14 (63.6%)
Other	1 (14.3%)	1 (14.3%)	2 (25%)	4 (18.2%)
Unanswered	1 (14.3%)	0	0	1 (4.5%)

^a^ HS = High school. ^b^ BA = Bachelor’s degree. * BMI differences between groups (*p* = 0.02).

**Table 3:** Pretest score, posttest score, and change score, inhibition for walking, standing, and sitting using the Stroop Color–Word Test.

Condition	Pretest Score	Posttest Score	Change Score
	Mean (SD)	Mean (SD)	Mean (SD)
	Range; Median	Range; Median	Range; Median
Walking	49.00 (8.27)	47.71 (5.94)	−1.29 (5.94)
	38–59; 49	39–55; 50	−9–6; −1
Standing	43.86 (5.76)	49.00 (6.27)	5.14 (5.90)
	35–52; 44	41–59; 48	−6–12; 7
Sitting	38.88 (7.42)	44.50 (7.21)	5.63 (5.04)
	23–46; 41	36–53; 45	−4–13; 7

Note. Change score is equal to posttest mean minus pretest mean. Higher change score = improvement.

**Table 4:** Percent change scores from pretest to posttest for inhibition and cognitive flexibility/shifting on the Stroop Color–Word Test and WCST for walking, standing, and sitting.

Variable	Walking	Standing	Sitting
Stroop Color–Word Test	−2.63%	11.72%	14.45%
WCST: total errors	5.86%	12.53%	4.91%
WCST: perseverative responses	9.64%	11.16%	6.34%
WCST: perseverative errors	9.97%	10.22%	5.14%
WCST: non-perseverative errors	0.25%	13.92%	3.39%
WCST: percent conceptual level responses	5.56%	13.51%	5.88%

Note. Percent change score is equal to posttest mean minus pretest mean divided by pretest mean multiplied by 100.

**Table 5:** Pretest score, posttest score, and change score in cognitive flexibility/shifting for walking, standing, and sitting using the Wisconsin Card Sorting Task.

WCST	Pretest Score	Posttest Score	Change Score
	Mean (SD)	Mean (SD)	Mean (SD)
	Range; Median	Range; Median	Range; Median
Walking			
Total errors	53.57 (11.37)	56.71 (12.01)	3.14 (6.59)
	35–65; 58	40–71; 60	−8–13; 4
Perseverative responses	51.86 (11.84)	56.86 (11.67)	5.00 (9.93)
	32–67; 55	43–72; 61	−8–21; 3
Perseverative errors	51.57 (12.2)	56.71 (12.66)	5.14 (10.56)
	33–67; 53	40–73; 61	−6–24; 1
Non-perseverative errors	55.43 (11.22)	55.57 (11.27)	0.14 (6.12)
	41–69; 55	41–70; 56	−9–11; 0
Percent conceptual level responses	54.00 (14.45)	57.00 (12.21)	3.00 (7.62)
	33–75; 58	39–70; 63	−8–14; 4
Standing			
Total errors	55.86 (9.79)	62.86 (7.34)	7.00 (11.24)
	39–70; 56	52–71; 64	−9–19; 10
Perseverative responses	55.00 (11.62)	61.14 (7.95)	6.14 (13.26)
	35–69; 57	49–72; 63	−12–22; 2
Perseverative errors	55.86 (11.20)	61.57 (7.81)	5.71 (12.45)
	37–71; 56	51–73; 63	−10–23; 3
Non-perseverative errors	53.29 (7.16)	60.71 (7.25)	7.43 (10.10)
	42–65; 54	51–70; 60	−9–16; 14
Percent conceptual level responses	57.14 (12.55)	64.86 (7.73)	7.71 (13.39)
	35–73; 58	52–73; 68	−11–24; 5
Sitting			
Total errors	53.38 (9.16)	56.00 (13.43)	2.63 (8.94)
	41–67; 53	35–76; 56	−10–13; 4
Perseverative responses	53.13 (8.18)	56.5 (10.28)	3.38 (8.48)
	41–65; 53.5	42–70; 56	−8–12; 7.5
Perseverative errors	53.5 (8.38)	56.2 (10.7)	2.75 (8.86)
	40–65; 54	41–71; 55	−9–12; 7
Non-perseverative errors	51.63 (9.2)	53.38 (13.85)	1.75 (8.8)
	37–63; 53.5	33–77; 55	−9–18; 1
Percent conceptual level responses	53.25 (9.42)	56.38 (16.42)	3.13 (10.6)
	41–62; 57	30–80; 56	−11–20; 1.5

Note. Change scores are equal to posttest scores minus pretest scores =. No *t*-scores were calculated for total correct, conceptual level responses, trials to complete first category, categories completed or failure to maintain set. Higher score = better performance.

## Data Availability

The data sets used for conclusions made in this research study are private and confidential due to ethical and legal requirements.
